# The philosophic origins of science and the evolution of the two cultures.

**DOI:** 10.3201/eid0601.000115

**Published:** 2000

**Authors:** N C Myrianthopoulos


### The Philosophic Origins of Science and the Evolution of the Two Cultures[1]

**Ntinos C. Myrianthopoulos**

"A small but growing number of American philosophers have opened private practices as 'philosopher practitioners' offering a therapy based on the idea that solutions to many personal, moral, and ethical problems can be found not in psychotherapy or Prozac but deep within the 2,500-year-old body of philosophical discourse."

This quotation from the New York Times of March 8, 1998, may have been startling to some and amusing to others—the New Yorker used it as a preamble to a humorous article—but not to anyone, myself included, who has enjoyed the pleasure of delving into the history of philosophy and who appreciates its relevance to the scientific process. What a splendid opportunity, then, to explore the philosophic origins of science and its long and fruitful legacy.

The quest for knowledge is an old preoccupation with roots in prehistory, starting with Adam and Eve, who did not go about it scientifically—and you know what happened to them. Even so, ancient humans continued the quest to understand and study the nature around them: the trees and the animals, the bearing of children, the heavenly bodies; that is, the natural phenomena that today we refer to as the natural sciences.

Among the old civilizations, the Babylonians and Egyptians contributed considerably to these sciences, practiced primitive medicine and surgery, and collected facts about natural history and biology. It was, however, left to the Greeks to enlarge the scope of these collections and formulate from the facts a unified concept of nature and the laws that govern it.

The oldest Greek thinkers were natural philosophers, and it was much later that ethical issues and other problems found a place in Greek thought. Practically all philosophers were teachers; many had their own schools, had to teach several subjects (rhetoric, ethics, poetics, astronomy, physics, biology), and wrote treatises on these subjects, which were surely sometimes used as textbooks. A few of these philosophers were also poets and wrote their own books in verse; Empedocles, for example, wrote two treatises, "On Nature" and "Purifications," in dactylic hexameter. I shall mention only one of these early philosophers, Democritos (approximately 450 BC), for two reasons. The first is that he was the very antithesis of the usual image of a brooding philosopher; he was known by several nicknames, one being "the laughing philosopher" because he was good humored and jolly all the time (γελουσινός). In fact, one of his noteworthy treatises is entitled "On Cheerfulness." The other reason is that to most of us Democritos is known mainly as the father of the atomic theory, which is not quite the case. It was his teacher, Leukippos, who first conceived and formulated the atomic theory, which Democritos immediately espoused and refined. He wrote on almost every field known at the time: physics, psychology, logic, astronomy, the senses, the mind, music, poetics. Aristotle thought highly of Democritos and refers to his work frequently, particularly in his various biological writings.

Aristotle was certainly the greatest of this genre of philosophers and is justly considered one of the greatest thinkers of all time. He was born in 384 B.C. in Stageira, Macedonia, where his father was the physician to the royal court. At age 17, he was sent to Athens to study with Plato in the Academy, where later he also taught. He spent 3 years in Asia Minor in the court of his former student Hermeias, who gave him his niece in marriage. He was then appointed by King Philip of Macedon to be the tutor of his impetuous and brilliant teenaged son Alexander, and after 3 years, he moved back to Athens. Now

[1]Aristotelian lecture given at the 9th International Clinical Genetics Seminar, July 4, 1998, Limassol, Cyprus. Parts of this lecture were published in "Cosmos, 1999," the Journal of the Cosmos Club of Washington, D.C.

a well-to-do man under the protection of Alexander, he founded his own school, the Lyceum (named after the nearby temple of Apollo Lycaeus). The Lyceum served as the prototype of a learned educational institution throughout the world. Here Aristotle taught while taking walks with his students and collaborators and wrote an incredible amount on various and different subjects, including logic, physics, ethics, art poetry, politics, economics, psychology, and biology. He retired to Chalkis, Euboea, where he died at age 62.

To the biologist, Aristotle's work on the "Generation of the Animals" is of special interest because it is the first systematic treatise on animal reproduction and embryology, taxonomy, and evolution. The "Generation of the Animals" is the culmination of Aristotle's zoological works that comprise 10 volumes and include "On the History of Animals," "On the Parts of Animals," and "On the Soul." I shall only touch upon those areas in which he made lasting contributions in the development of the biological sciences that have come down to us.

Aristotle repeatedly pointed out that his predecessors' work and conclusions were often marred by insufficient observation. He himself, after a remarkable analysis of the reproduction of bees, states that he cannot arrive at certain conclusions because "the facts have not yet been sufficiently ascertained. And if at any future time they are ascertained, then credence must be given to the direct evidence rather than to the theories; and to the theories also, provided that the results which they show agree with what is observed." This, indeed, is the principle upon which his work is based. It is also the definition of the scientific method, which was later broadened in scope, especially by Bacon, and by and large constitutes the basis of the scientific method we practice today. Note the subtle yet critical point: Aristotle does not say "the results prove the theory," but "the results agree with the observations." Today, we take this reasoning for granted, that science proceeds and progresses not by proving hypotheses, but by disproving them. If the observations do not agree with a hypothesis, we shelve it; if it does agree with a high enough level of certainty and consistent repetition of the results, we accept it, but we can never prove it.

Up to the time of Aristotle, there had been no serious attempts at classification of animals. Thus, his classification was based almost entirely on his own observations. For animals not found in Greece, he referred to credible observations by others, e.g., Herodotos. In this area also, Aristotle made very important contributions by characterizing and differentiating among a number of systematic categories. In his own words, "Animals may be characterized according to their way of living, their actions, their habits, and their bodily parts." The most important criterion is certainly the parts of the animals, both external and internal: organs of movement, respiration, sense, blood circulation. By combining various qualities, he defined and characterized the groups. Aristotle's two major categories are blooded animals (he refers to red blood only) and bloodless animals.

Under blooded animals: humans, viviparous quadrupeds, oviparous quadrupeds, and footless animals (reptiles, amphibians), birds, and fishes.

Under bloodless animals: malacostraca (soft-shelled, crustacea); malakia (soft, without shell, cephalopods); entoma (insected animals, insects); and ostrakoderma (shell-skinned, testacea). These categories and nomenclature are still used today.

Aristotle also classified animals according to their mode of reproduction, but the most important part of his classification is the final two categories, the genus (γένος) and the species (είδος), the latter referring to the individual animal form: horse, dog, lion. This is a farsighted classification, and though it cannot be compared with the Linnaean with its manifold categories, it is certainly a pioneering achievement.

In his work on the reproduction of animals, Aristotle differentiates sexual and asexual reproduction. In sexual reproduction, male and female contribute equally, and in his thorough investigation of the development of animals from egg and embryo, Aristotle points out the phenomenon that we know today as "ontogeny recapitulates phylogeny." He disagreed sharply with the opinion of earlier philosophers that the seed is derived from all parts of the body and thus gives rise to similar individuals. On the contrary, he asserts that the seed goes to all parts of the body to form an individual, an explanation shown to be correct 2,000 years later. In addition, during embryonic development, there is a specific movement or substance in each part of the body which brings about its development as a specific part of the embryo. Today, we call such substances organizers. Aristotle also recognized congenital malformations as imperfect developmental events in the embryo due to various causes, one being some irregularity in the seminal fluid. He correctly understood the functions of the placenta and the umbilical cord and was an ardent supporter of epigenesis. He made his observations in several animals, and it can be said that he introduced the comparative method to embryology.

Aristotle has been called the first evolutionist. His theory of evolution lies not only in the sphere of discovery, but also in his system of thought, embracing all phenomena of life. Here we find enunciated for the first time a truly complete theory of evolution, subject to natural laws and progressing from the lower to the higher forms of being. Although partly based on metaphysical speculation, the theory has proven fertile ground for future biologists.

Aristotle constantly compares nature and the products of nature with art and the products of art. Like nature, the artist or craftsman works to produce a finished product. Like the artist, nature uses instruments charged with specific modulations to bring these products to fulfillment. The most typical of these products of nature are, of course, living creatures. Nature aims always to produce a finality in the sense of a completely formed individual and that is the Final Cause in each case. "There is," Aristotle says, "more beauty and purpose found in the works of nature than in those of art." And who can disagree?

Although Aristotle was not the last of the era in which the study of nature was in the province of philosophy, by the time of his death, there were already signs of specialization, that is, philosophers began to be concerned mainly with ethics and metaphysics, leaving the other subjects to those more informed about them. This trend continued through the Hellenistic and Roman times. Later, with the increase of knowledge and ease in its dissemination, the establishment of libraries, and invention of the printing press, the graduates of schools of higher education came to be recognized either as scientists, biological or physical, or as artists, poets, writers, painters, or musicians and received different credentials, in our time Bachelor of Arts or Bachelor of Science. In spite of the widening schism, the philosophic origin of the sciences and the arts is acknowledged and maintained today in the award of the highest academic degree, that of Doctor of Philosophy.

In his controversial Rede lecture presented in Cambridge in 1959, C.P. Snow (1905-1980), the British intellectual, first used the phrase "The Two Cultures" to describe the world of the sciences and the world of the arts, which had become culturally isolated. Sir Charles Percy Snow was himself a distinguished physicist, who during World War II made significant contributions to the British and Allied war effort and for his services was elevated to the peerage. He was also an excellent novelist. His magnum opus, "Strangers and Brothers," comprises eleven volumes written over 30 years, in which he recounts a saga of lives, events, and the passage of time, both for individuals and for English society as a whole. As Snow himself described his existence, "There have been plenty of days when I have spent the working hours with scientists and then gone off at night with some literary colleagues. I mean that literally. It was through living with these groups and much more, I think, through moving regularly from one to the other and back again that I got occupied with the problem of what, long before I put it on paper, I christened to myself as 'the two cultures.' For constantly I felt I was moving among two groups—comparable in intelligence, identical in race, not grossly different in social origin, earning about the same incomes, who have almost ceased to communicate at all, who in intellectual, moral and psychological climate had so little in common, that instead of going from Burlington House or South Kensington to Chelsea, one might have crossed an ocean."

Snow, of course, was addressing a situation prevalent in England and Europe in general in the late 1950s, but at that time, conditions on this side of the Atlantic may have been a little better. Nevertheless, the two cultures still exist and combine infrequently in rare and exceptional individuals.

Every era has had such exceptional individuals. The Renaissance produced a sprinkling of them, the towering and awesome figure of that era being Leonardo da Vinci (1452-1519), the Italian painter par excellence, and sculptor, but also architect, engineer, musician, inventor, anatomist, physiologist, geologist, botanist, and everything that you can imagine, and many things you cannot. Indeed, a thumbing through his voluminous diaries, originally

